# Concerns about Continuing Claims that a Protein Complex Interacts with the Phosphorelay

**DOI:** 10.1128/mBio.03371-19

**Published:** 2020-03-10

**Authors:** Richard Losick

**Affiliations:** a Department of Molecular and Cellular Biology, Harvard University, Cambridge, Massachusetts, USA; University of Minnesota Medical School

**Keywords:** *Bacillus subtilis*, RNase Y, phosphorelay

## LETTER

The major endonuclease for mRNA decay in Bacillus subtilis is the integral membrane protein RNase Y. We have shown that RNase Y interacts directly with a widely conserved, three-protein complex, the Y-complex, that is required for the majority of RNase Y-mediated mRNA cleavage events in B. subtilis (1, 2). This letter raises concerns about a recent paper in *mBio* (3) and three preceding publications (4–6) that argue that the Y-complex has an additional, add-on function in which it directly interacts with two proteins (Spo0F and Spo0B) in a phosphorelay that is responsible for phosphorylating the master regulatory protein Spo0A.

That the Y-complex interacts with RNase Y is based on robust two-hybrid data with multiple positive and negative controls (1), pulldown experiments done independently by the authors (4) and by us (1), our demonstration that the Y-complex associates with the membrane in an RNase Y-dependent manner (2), and our demonstration that mutations of the Y-protein genes have global effects on RNA levels, largely matching the effects of mutations of the RNase Y gene itself (2).

We have also shown that the Y-protein genes are widely conserved among *Firmicutes* (being coconserved with the RNase Y gene), including, importantly, many bacteria that lack the Spo0F-Spo0B-Spo0A phosphorelay (1). Finally, we have shown that a Y-gene mutation not only blocks processing of a canonical RNase Y substrate in B. subtilis but also in Staphylococcus aureus, which lacks the phosphorelay (2).

In light of the evidence that the Y-complex is a broadly conserved accessory factor for RNase Y, the authors’ proposal (3) that the complex has been “coopted” to have an additional function in B. subtilis, directly interacting with Spo0F and Spo0B (which neither resemble each other nor RNase Y) to stimulate phosphorylation of Spo0A, seems improbable. Nonetheless, it is a formal possibility. However, when something is unlikely, the bar is raised for providing convincing evidence. The claim that the Y proteins directly interact with the relay has weaknesses. First, the claim is based on phosphorelay experiments showing only a twofold stimulation of activity, and the interpretation of this activity is difficult because the primary data showing phosphorylated relay proteins were not presented. Moreover, the stimulation experiments lacked the control of testing other proteins, such as other proteins containing Fe-S clusters. Second, the only data supporting a direct interaction with relay proteins is a two-hybrid experiment in which only one of the Y proteins exhibited a signal with Spo0F and Spo0B and that signal was fourfold lower than that for self-interaction between Y proteins (4, 5). In contrast, we have published based on a system showing robust interactions of Y proteins with each other and with RNase Y that we detected no interaction with Spo0B (1). In addition, and importantly, pulldown experiments done independently by the authors (4) and by us (1) revealed RNase Y, but not Spo0F or Spo0B. Finally, the highly pleiotropic effects of Y mutations, such as cleaving the polycistronic mRNA for glycolytic enzymes and causing elevated levels of mRNAs involved in nitrogen metabolism (2), undermine claims attributing *in vivo* effects on Spo0A activity as being due to direct interactions with the relay. They are instead most easily explained as being due to indirect and global effects on the transcriptome and metabolism. To be clear, the primary basis for these concerns is not the current *mBio* paper (3) but rather the data reported in earlier publications (4, 5).

In the 7 years since the authors’ claims were first made (4), no biochemical evidence confirming that any of the Y proteins or the three-protein complex bind to Spo0F and/or Spo0B, including the present report, has been published. In sum, and to resolve this disagreement, I request that the proponents of the view that Y proteins function by “direct interactions” provide compelling evidence that the Y-complex binds to Spo0F and Spo0B by any of the standard methods for demonstrating protein-protein interactions. If it is not possible to obtain such evidence, I request that we come to an agreement that a second add-on function for the complex is at best speculative.
